# Functional communication ability in frontotemporal lobar degeneration and Alzheimer’s disease

**DOI:** 10.1590/S1980-57642009DN20100007

**Published:** 2008

**Authors:** Isabel Albuquerque M. de Carvalho, Valéria Santoro Bahia, Leticia Lessa Mansur

**Affiliations:** 1 PhD, Speech-language Pathologist of Old Age Research Group (PROTER) and CEREDIC, School of Medicine, University of São Paulo. São Paulo , Brazil.; 2 MD, PhD. Behavioral and Cognitive Neurology Unit, Department of Neurology, Hospital das Clínicas, University of São Paulo School of Medicine, São Paulo, Brazil.; 3 PhD, Assistant Professor - Department of Physiotherapy, Speech Therapy and Occupational Therapy – University of São Paulo. São Paulo, Brazil.

**Keywords:** communication, functional, language, assessment, dementia, comunicação, funcionalidade, linguagem, avaliação, demência

## Abstract

Functional communication is crucial for independent and efficient communicative behavior in response to every day activities. In the course of dementia progression, cognitive losses may impair these abilities. For this reason, functional communication assessment should be part of a formal assessment to quantify and qualify the impact of deficiency on patients’ lives.

**Objective:**

To compare functional communication abilities in fronto-temporal lobar degeneration (FLTD) and Alzheimer’s disease (AD).

**Methods:**

Six AD patients (mean age: 82.50±2.66 years; mean education: 5.67±3.61 years), and eight FTLD patients (mean age: 57.13±9.63 years; mean education: 10.86±6.91 years) had their close relatives answer the Functional Assessment of Communication Skills for Adults (Asha-facs) . Statistical analyses correlated the performance on each of the Asha-facs domains (social communication, communication of basic needs; reading, writing, number concept and daily planning) between both groups.

**Results:**

Analyses showed that functional communication was similar for AD and FTLD patients. Only two items had statistical difference, namely ‘Comprehension of inference’ (AD 6.7±1.33; FTLD 2.43±2.30, p=0.017) and ‘capacity to make basic money transactions’ (AD 2.17±2.04; FTLD 4.00±0.90, p=0.044). Comparison among the four domains’ mean scores revealed no significant difference.

**Conclusion:**

The Asha-facs is a useful instrument to characterize functional communication abilities in both FTLD and AD. Nevertheless, the analysis presented for this sample showed that the Asha-facs could not discriminate which aspects of the FTLD and AD differed.

## Abstract (pt)

Comunicação funcional é fundamental para a independência e eficiência comunicativa em resposta à demanda do dia-a-dia. Ao longo do processo demencial, déficits cognitivos podem comprometer tais habilidades. Assim, a avaliação das habilidades funcionais de comunicação deve fazer parte do protocolo de avaliação para quantificar e qualificar o impacto da deficiência na vida do paciente. Comparar as habilidades funcionais de comunicação em pacientes com degeneração lobar fronto-temporal (DLFT) e doença de Alzheimer (DA).

**Métodos:**

Foram avaliados seis familiares próximos de pacientes com diagnóstico de DA e oito familiares próximos de pacientes com diagnóstico de DLFT. Os familiares responderam à Avaliação Funcional das Habilidades de Comunicação - Asha-facs sobre o comportamento cognitivo-comunicativo dos pacientes. Análise estatística comparou o desempenho dos dois grupos em cada domínio do Asha-facs: comunicação social, comunicação de necessidades básicas, leitura, escrita e conceitos numéricos e planejamento diário.

**Resultados:**

A habilidade de comunicação funcional foi similar para pacientes com DA e com DLFT. Apenas dois itens apresentaram significância estatística: ‘compreensão de inferências’ (DA 6,7±1,33; DFT 2,43±2,30, p=0,017) e ‘capacidade para fazer transações básicas com dinheiro’ (DA 2,17±2,04; DFT 4,00±0,90, p=0,044). A comparação da pontuação média dos quarto domínios não apresentou diferença signitficativa.

**Conclusão:**

O instrumento Asha-facs mostrou-se útil na caracterização das habilidades funcionais e comunicação para pacientes com DA e com DLFT. No entanto, a análise apresentada sugere que a Asha-facs pode identificar tais déficts, mas não verificar quais aspectos diferenciam pacientes com DLFT e DA.

Functional communication is the ability to receive or convey a message as well as to communicate effectively and independently in a natural environment regardless of the mode of communication.^1^

This definition embraces an integrated concept of communication rather than isolated processes. It encompasses any verbal or non-verbal communication modality and considers efficiency and independence as essential to an appropriate response to everyday demands.^2^

Communication may be impaired from the first stage of dementia.^3-6^ Consequently, speech and language evaluation should assess the ability to communicate in different situations, independently of speech, language or cognitive impairment. This assessment should consider environmental modifications, use of hearing aids, time needed to communicate and behaviors that may interfere with communicative ability in an ecological situation.

Such assessment may be better understood considering the International Classification of Functioning, Disability and Health (WHO/ICF), which considers ‘body’ as functions of body systems or body structures, and ‘activity and participation’ as a complete range of domains denoting aspects of functioning from both an individual and environmental perspective. ‘Activity’ is defined as the execution of a task or action by an individual and ‘participation’ as the involvement in a life situation. The contextual factors represent the complete background of an individual’s life and living which may have an impact on the individual in good health.^7^

Based on this model, functionality focuses on components of body structure/function; activity/participation, and environmental/personal factors used in a positive way.

Functional activity assessment scales center on quantifying and qualifying the deficiency caused by the disease from the viewpoint of functionality. They also facilitate therapeutic planning and familial/caregiver orientation.^8^

The Asha-facs enhances traditional assessment of speech, language and cognitive deficits, with information on deficit effects in the daily cognitive-communicative context.

This study aimed to compare two types of dementia: Frontotemporal Lobar Degeneration (FTLD) and Alzheimer’s disease (AD).

Alzheimer’s disease is a highly prevalent type of dementia^9^ with a predominant memory deficit followed by another cognitive deficit. Functional analyses of communication in healthy elderly and those with AD may suggest that important communication impairment throughout the disease worsens patient’s independence and autonomy, in addition to compromising their quality of life.^10^

FTLD involves the frontal and anterior temporal lobes deficits. It is characterized by prominent and gradual behavioral and language disorders, whereas memory is relatively preserved.^11,12^

Neary et al. (1998)^11^ distinguished three variants of FTLD: the frontal variant of frontotemporal dementia (FTD), semantic dementia (SD) and progressive non-fluent aphasia (PNFA). FTD is the most common clinical presentation among them, accounting for approximately half of all FTLD diagnoses. The characteristic features include loss of insight, disinhibition, impulsivity, apathy, reduced empathy for others, poor self care, stereotypic behavior, emotional blunting, and changes in eating patterns.^13,14^

PNFA is a form of FTLD with a language component, and a reduction in spontaneous discourse, phonemic paraphasias and preserved comprehension. SD is characterized by the loss of semantic associations while other language aspects remain preserved.^15^

## Methods

A total of 14 subjects, 6 relatives of AD patients and 8 relatives of FTLD patients participated in the study. For the AD group, relatives were consort (2); sons (3) and sister (1). For the FTLD group, the relatives were daughter-in-law (1); brother (1); consort (5) and daughter (1).

The AD group consisted of individuals who met the criteria for probable AD according to the National Institute of Neurological and Communicative Diseases and Stroke/Alzheimer’s Disease and Related Disorders Association - NINCDS-ADRDA,^16^ and were all on anticholinesterasic treatment.

The FTLD group had diagnoses based on anamneses, neurological examination, and neuropsychological assessment, structural neuroimaging (CT or MRI) and functional SPECT imaging along with a battery of routine screening blood tests. Among the 8 FTLD patients, 6 were diagnosed with FTDs, one with PNFA and one SD.

All subjects were selected at the Behavioral and Cognitive Neurology Unit of Hospital das Clínicas, in São Paulo, Brazil.

The Asha-facs is a functional scale that assesses a complex communication situation in an ecological environment. It consists of a communicative independence score and qualitative dimensions of communication scores. The Asha-facs communication independence scale is composed of 43 items divided into four domains: Social Communication (21 items); Communication of Basic Needs (7 items); Reading, Writing and Number Concepts (10 items); and Daily Planning (5 items). Within each domain, functional behaviors are observed and rated. The 7-point Scale of Communication Independence measures functional communication performance along a continuum of independence, in terms of levels of assistance and/or prompting needed in order to communicate. The Asha-facs maximum score is 7, which means that the patient is totally independent to perform the communication behavior; 6 indicates the patient rarely needs assistance; 5 that he/she needs assistance occasionally; 4 means he/she needs moderate assistance; 3 that he/she needs assistance very frequently; 2 means that patient needs constant assistance to perform a communicative behavior and 1 means that the patient is not able to perform the activity even with all assistance provided. The scale can be administered in approximately 20 minutes.

The Asha-facs is previously validated for the Brazilian population with AD.^17^

The family answered the Asha-facs about the subject being tested. Descriptive analyses were carried out (means and standard deviation) of socio-demographic variables and descriptive data. Statistical analyses were performed to compare the performance in both groups on each Asha-facs domain. All participants signed the informed consent forms.

## Results

Socio-demographic characterization showed equivalence in terms of education for both groups, mean years of education for AD (5.67±3.61 years) and for FTLD (10.86± 6.91). There was a significant difference of age (p<0.002) between groups (AD: 82.50±2.66; FTLD: 57.13±2.66), which was expected due to the nature and characteristics of the diseases. The MMSE mean score for the AD group was 12 (±6.9) and for FTLD was 17.50 (±11.2).

The family answered the Asha-facs scale about the subject being tested.

Analyses showed that functional communication was similar for AD and FTLD patients. Only two items had statistical difference which was ‘Comprehension of inference’ (AD 6.7±1.33; FTLD 2.43±2.30, p=0.017) (Table 2) and ‘capacity of making basic money transactions’ (AD 2.17±2.04; FTLD 4.00±0.90, p=0.044) (Table 4). The comparison of the four domains’ mean scores revealed no significant difference. For this sample the Asha-facs was not able to differentiate patients between one dementia diagnosis or another, although it was possible to identify some patterns of communication behavior that were more common in one or other type of dementia or the other. AD and FTLD patients have different communication complaints although their ability to perform communication is low in any case.

**Table 2:** Comparison of FTLD and AD performance on each item of social communication domain.

Social communication	FTLD (n=8)	AD (n=6)	p
1. Refers to familiar people by name	5.00 (±2.20)	6.33 (±1.03)	0.282
2. Requests information of others	4.63 (±2.67)	5.50 (±2.51)	0.615
3. Explains how to do	3.25 (±1.98)	4.50 (±2.59)	0.391
4. Expresses agreement/disagreement	6.00 (±1.77)	6.50 (±1.22)	0.518
5. Exchanges information on the phone	2.63 (±2.45)	5.20 (±2.17)	0.071
6. Participates in a group conversation	4.00 (±2.62)	4.33 (±2.25)	0.894
7. Answers yes/no questions	6.00 (±2.14)	5.67 (±2.16)	0.808
8. Follows simple verbal directions	4.88 (±2.47)	4.50 (±2.59)	0.735
9. Understands intent	3.38 (±2.62)	4.50 (±2.95)	0.543
10. Smiles or laughs at lighthearted comments	4.00 (±2.78)	6.17 (±2.04)	0.113
**11. Understands non-literal meaning and inference**	**2.43 (±2.30)**	**6.17 (±1.33)**	**0.017**
12. Understands conversations when they occur in noisy or distracting situations	3.88 (±2.23)	4.50 (±1.76)	0.587
13. Understands what's heard on TV and radio	4.13 (±2.53)	4.33 (±2.16)	0.948
14. Understands facial expressions	3.75 (±3.01)	6.83 (±0.41)	0.060
15. Understands tone of voice	6.25 (±2.12)	6.83 (±0.41)	0.999
16. Initiates communication with other people	4.75 (±2.55)	6.17 (±2.04)	0.152
17. Adds new information on a topic in a conversation	3.63 (±2.39)	4.67 (±1.97)	0.349
18. Changes topics in conversation	5.00 (±2.78)	4.83 (±2.71)	0.829
19. Adjusts to a change in topic by conversational partner	3.50 (±2.51)	3.83 (±2.23)	0.740
20. Recognizes his/her own communication errors	3.25 (±2.66)	3.83 (±2.71)	0.738
21. Corrects his/her own communication errors	3.00 (±2.77)	4.00 (±2.53)	0.711
Total	4.19 (±1.64)	5.21 (±1.37)	0.175

FTLD: frontotemporal lobar degeneration; AD: Alzheimer's disease; p<0.05

**Table 4:** Comparison of FTLD and AD performance on each item of reading, writing and number concepts domain.

Reading, writing and number concepts	FTLD (n=8)	AD (n=6)	p
29. Understands simple signs	4.13 (±2.64)	3.33 (±2.42)	0.507
30. Uses common reference materials (e.g., telephone book, TV guide)	2.13 (±2.23)	3.00 (±3.10)	0.684
31. Follows written directions	2.88 (±2.59)	2.80 (±1.79)	0.940
32. Understands basic printed material (e.g., menus, headlines)	3.50 (±2.98)	4.83 (±2.99)	0.575
33. Prints/writes/types name	6.00 (±2.14)	6.67 (±0.82)	0.719
34. Fills out short forms	4.25 (±2.55)	3.00 (±2.76)	0.386
35. Writes messages (e.g., "Call your mother")	2.29 (±2.36)	3.00 (±3.10)	0.792
36. Understands signs with numbers (e.g., price tags, speed limit signs)	5.13 (±2.10)	4.00 (±3.29)	0.734
**37. Makes basic money transactions (e.g., pays for items at grocery store, recognizes when given the wrong change)**	**4.00 (±0.93)**	**2.17 (±2.04)**	**0.004**
38. Understands simple units of measurement (e.g., weights, distances, quantities in recipes)	4.00 (±2.31)	2.67 (±2.42)	0.374
Total	3.83 (±1.61)	3.61 (±1.76)	0.846

FTLD: frontotemporal lobar degeneration; AD: Alzheimer's disease; p<0.05

## Discussion

It is known that difficulty in communicating is understood as deterioration in functionality, which leads to increased dependence. This becomes a very important issue when we address functionality in dementia diagnosis. Both AD and FTLD patients will develop, at some point, communication difficulties which will cause loss of independence.

The Asha-facs is a simple, quick and low-cost assessment that provides information on the patient’s cognitive-communicative behavior in their environment. Despite the fact that the results are still preliminary due to the small sample, some important data emerged regarding communication deficits in dementia processes.

Data on language deficits in the literature point to heterogeneous deficits of this cognitive function.^3,4,18-21^ One possible explanation could be the fact that interpersonal communication occurs through language and its interface with other cognitive functions, impaired in dementia, such as memory, attention and executive function.

Functional communication evaluation yields three important findings about the way in which an individual deals with their own living environment, albeit socially or occupationally related, through the investigation of their communication independence in each Asha-facs domain.

Within the four domains composing the scale, Social Communication was observed as the most preserved in dementia of Alzheimer’s type. Even though there was a significant difference in only one item of the domain, the average score of AD groups seemed to be slightly higher compared to FTLD groups. An explanation that can be raised is that AD patients probably compensate for any difficulties in communicative interaction, using clues from the interlocutor to fill in possible communicative gaps in the discourse, or, there could be self-monitoring with surrounding support to facilitate communication. Thus, it seems that natural compensation is still observed in the initial phase of AD. On the other hand, it seems that FTLD patient have a higher behavioral variance than AD, where at times when FTLD patients experience apathy or agitation, this kind of compensation becomes more difficult to process.

In the Communication of Basic Needs domain, the results of both groups were observed to be quite similar, being relatively preserved for this population. The Reading, Writing and Numerical Concepts domain seems to be composed by items sensitive to the dementia process for either AD or FTLD, as they were impaired in both groups. The item “make basic money transactions” was significantly different since FTLD patients had better performance on this item than AD patients, probably because the latter have less memory issues and more calculus deficits that would interfere in this skill than AD patients. The Daily Planning domain also had a similar mean score for both groups, showing an important decline in performance for the behaviors proposed.

The comparisons made in this study showed that functional deficit was observed in AD and FTLD dementia, mainly in the domains of Social Communication and Reading, Writing and Numerical Concepts.

The results outlined above suggest that, even though we have no control group of normal elderly to compare the Asha-facs performance, another study^10^ carried out this comparison between normal elderly and AD patients and showed a significant decline for AD. Our study showed a similar performance for both groups, so we could infer that there is a similar difference between FTLD and normal elderly.

For example, almost all individuals presented some difficulty in “understanding conversation in noisy areas”, “using reference manuals”, “filling in a form”, “taking message notes” and “meeting scheduled commitments”.

The difficulties of functional communication corroborate the findings in the literature that characterize the heterogeneity of language deficit in dementia.

These results are still preliminary, but suggest an important panorama of language and communication deficits pertaining to the dementia process in AD and FTLD. More patients are being added to this sample for a broader study to verify the proposed hypotheses.

Indirect assessment through family members reflects their view of the patient and it is important to take into consideration the fact that they may pay more attention to the behavior alterations and incapacity suggesting that the patient is worse functionally than he/she really is. On the other hand this indirect assessment of functional evaluation scale predicts deficits and abilities in an ecological analysis while minimizing the patient’s exposure to long and exhausting cognitive testing. It is important to highlight that this evaluation quantifying and qualifying the deficiency caused by the disease in terms of functionality and is very important for patient follow-up, therapeutic planning and familial/caregiver orientation.

Although a deficit of functional communication ability in both AD and FTLD is known, the analysis presented for this sample showed that the Asha-facs could not discriminate which aspects of FTLD and AD differ, but was able to provide a profile of functional communicative deficit.

## Figures and Tables

**Table 1:** Socio-demographic characteristics.

	FTLD (n=8) Mean±SD	AD (n=6) Mean±SD	p
Age*	57.13±9.63	82.50±2.66	**0.002**
Education*	10.86±6.91	5.67±3.61	0.245
MMSE	17.5±11.20	12.00±6.90	0.44

* in years; FTLD: frontotemporal lobar degeneration; AD: Alzheimer's disease; p<0.05; MMSE: Mini-Mental State Exam.

**Table 3:** Comparison of FTLD and AD performance on each item of communication of basic needs.

Communication of basic needs	FTLD (n=8)	AD (n=6)	p
22. Recognizes familiar faces	6.25 (±1.39)	6.83 (±0.41)	0.653
23. Recognizes familiar voices	5.00 (±2.83)	6.50 (±1.22)	0.331
24. Makes strong likes or dislikes known	5.63 (±2.56)	6.00 (±2.45)	0.857
25. Expresses feelings (e.g., happy, sad)	4.50 (±2.07)	6.00 (±2.45)	0.127
26. Requests help when necessary	4.50 (±2.83)	5.00 (±3.10)	0.828
27. Makes needs or wants known	5.38 (±2.07)	5.50 (±1.64)	0.889
28. Responds in an emergency (e.g., calls 911)	1.50 (±1.51)	2.50 (±1.73)	0.393
Total	4.68 (±1.45)	5.58 (±1.50)	0.332

FTLD: frontotemporal lobar degeneration; AD: Alzheimer's disease; p<0.05

**Table 5:** Comparison of FTLD and AD performance on each item of daily planning.

Daily planning	FTLD (n=8)	AD (n=6)	p
39. Knows what time it is	4.88 (±2.03)	4.50 (±2.95)	0.999
40. Dials telephone numbers	5.63 (±1.92)	3.80 (±2.77)	0.275
41. Keeps scheduled appointments	2.38 (±1.77)	2.00 (±1.55)	0.680
42. Uses a calendar for time-related activities (e.g., scheduling, planning	1.88 (±1.81)	2.50 (±2.51)	0.686
43. Follows a map (e.g., finds a street on a road map)	2.00 (±2.00)	1.00 (±0.00)	0.287
Total	3.45 (±1.45)	2.85 (±1.79)	0.438

FTLD: frontotemporal lobar degeneration; AD: Alzheimer's disease; p<0.05
